# Whose Ear?: Proposal to conserve the name *Auricularia auricula* (L.) Underw. for *Auricularia auricula-judae* (Bull.) Quél.

**DOI:** 10.12688/f1000research.134821.1

**Published:** 2023-08-07

**Authors:** Kendra C. Autumn, Bryn T. M. Dentinger

**Affiliations:** 1 The Natural History Museum of Utah, Salt Lake City, Utah, 84108, USA; 2 School of Biological Sciences, The University of Utah, Salt Lake City, Utah, 84112, USA

**Keywords:** mycology, taxonomy, nomenclature, historical mycology, Jew's ear, jelly ear

## Abstract

*Auricularia auricula-judae* is a saprobic European jelly fungus with traditional culinary and medicinal significance, often said to resemble a human ear. It was originally named *Tremella auricula* by Linnaeus and has been moved to different genera since, but its specific epithet was also changed from *auricula* to *auricula-judae *by Bulliard in 1789, which is not normally a valid nomenclatural alteration. However, due to the practice of “name sanctioning” in the mycological nomenclatural code, this change has been accepted. This article outlines the nomenclatural and cultural history of the controversial name *Auricularia auricula-judae* and suggests its return to the original specific epithet *auricula*, as well as the designation of an epitype specimen.

## Introduction

The jelly fungus *Auricularia auricula-judae* (Bull.) Quél. (Auriculariaceae, Auriculariales, Agaricomycetes, Basidiomycota) is a saprobic mushroom that grows on multiple hardwood species and has a long history of medicinal and culinary use ( Kout and Wu 2022). Previously, various *Auricularia* specimens from Europe, North America, and Asia were regarded as belonging to a single species, *A. auricula-judae*, but molecular systematics have shown that European specimens form a distinct monophyletic clade ( Wu *et al*. 2015, 2021) *.* To reflect these findings, true *A. auricula-judae* are considered to occur solely in Europe, although similar *Auricularia* species have garnered interest both traditionally and in modern times as food and medicine, particularly in Asia.

*A. auricula-judae* has several common names: Judas’ ear, Jew’s ear, wood ear, tree ear, or jelly ear. This profusion of common names may represent awkwardness around the specific epithet. David Arora’s popular foraging-focused book *All That the Rain Promises and More…* uses an invalid species name, *Auricularia auricula*, removing *-judae* from the epithet, and offers the common name “Wood Ear”, with “Tree Ear” and “Judas’ Ear” as alternatives ( Arora 1991). Similarly, the 1975 *A Field Guide to Western Mushrooms* lists only Auricularia auricula ( Smith 1975). It’s unclear whether this habit of shortening the epithet is simply a response to the bulky hyphenation, or of the resemblance of the term “judae” to “Judaism” and other words associated with the Jewish people and religion. This superficial resemblance may have resulted in the common name Jew’s ear, or a reference to Jews may have been purposeful from the creation of the current epithet.

## History

**Figure 1.**
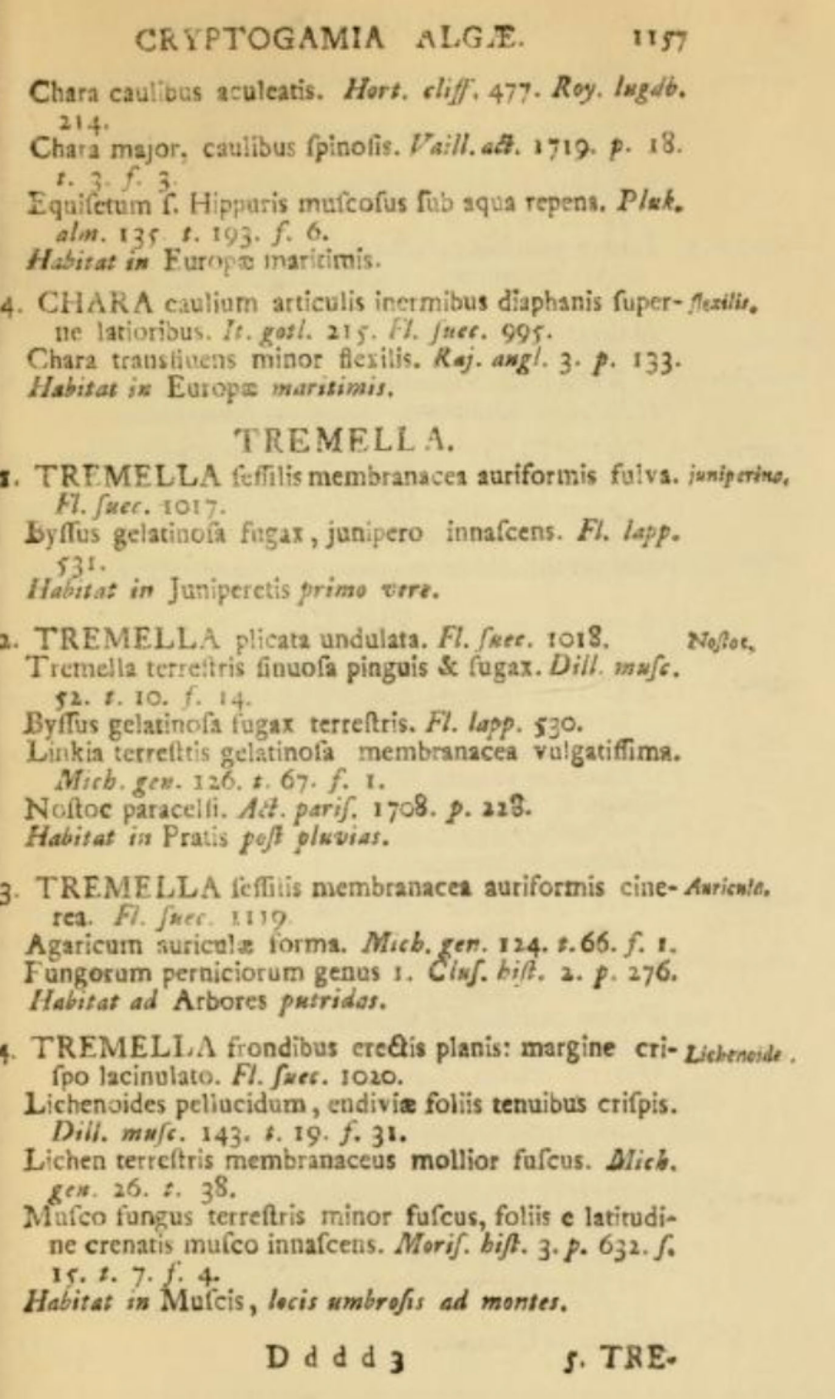
*Tremella auricula* erected in Linnaeus’ *Species Plantarum* (1753).

**Figure 2.**
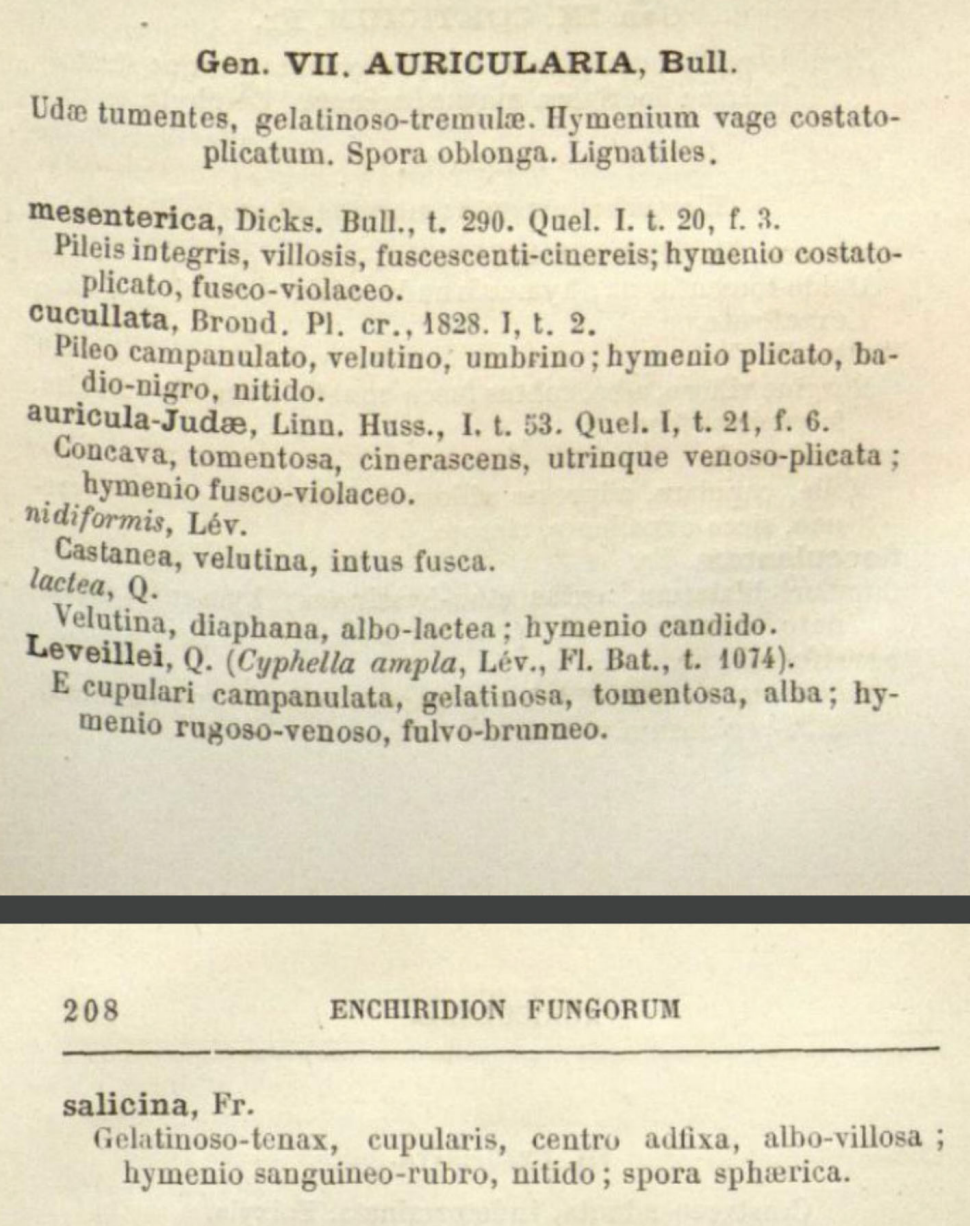
From *Enchiridion fungorum in Europa media et praesertim in Gallia vigentium*, Lutetiae, O. Doin (1886).

The binomial *Auricularia auricula-judae* emerged from a string of name changes during the 18th and 19th centuries. Linnaeus created the species *Tremella auricula* in 1753, as documented in *Species Plantarum* ( Figure 1) ( Linné 1753). In 1789, Bulliard appended “ *-judae”* to the specific epithet in his publication *Herbier de la France*, functionally changing the name to *Tremella auricula-judae*, an invalid alteration in regard to taxonomic convention ( Herbier de la France 1780-93). In 1822, Fries moved the species to the genus *Exidia* in the second volume of Systema mycologicum ( Fries 1821), preserving Bulliard’s specific epithet and creating the name *Exidia auricula-judae.* The current name *Auricularia auricula-judae* was established by Quélet in 1886 and recorded in *Enchiridion fungorum in Europa media et praesertim in Gallia vigentium* ( Figure 2) ( Quélet 1886).

Although Bulliard’s change to the specific epithet has no basis in the general rules of taxonomic naming, it was solidified by its acceptance by Fries, for whom there is a special provision in Article F.3 of the *International Code of Nomenclature for algae, fungi, and plants* ( May *et al*. 2019). Because of this exception, the publication of *Exidia auricula-judae* in *Systema mycologicum “*sanctions” the epithet *auricula-judae* and excepts it from the nomenclatural principle that would dictate its return to the original *auricula* as designated by Linnaeus.

**Figure 3.**
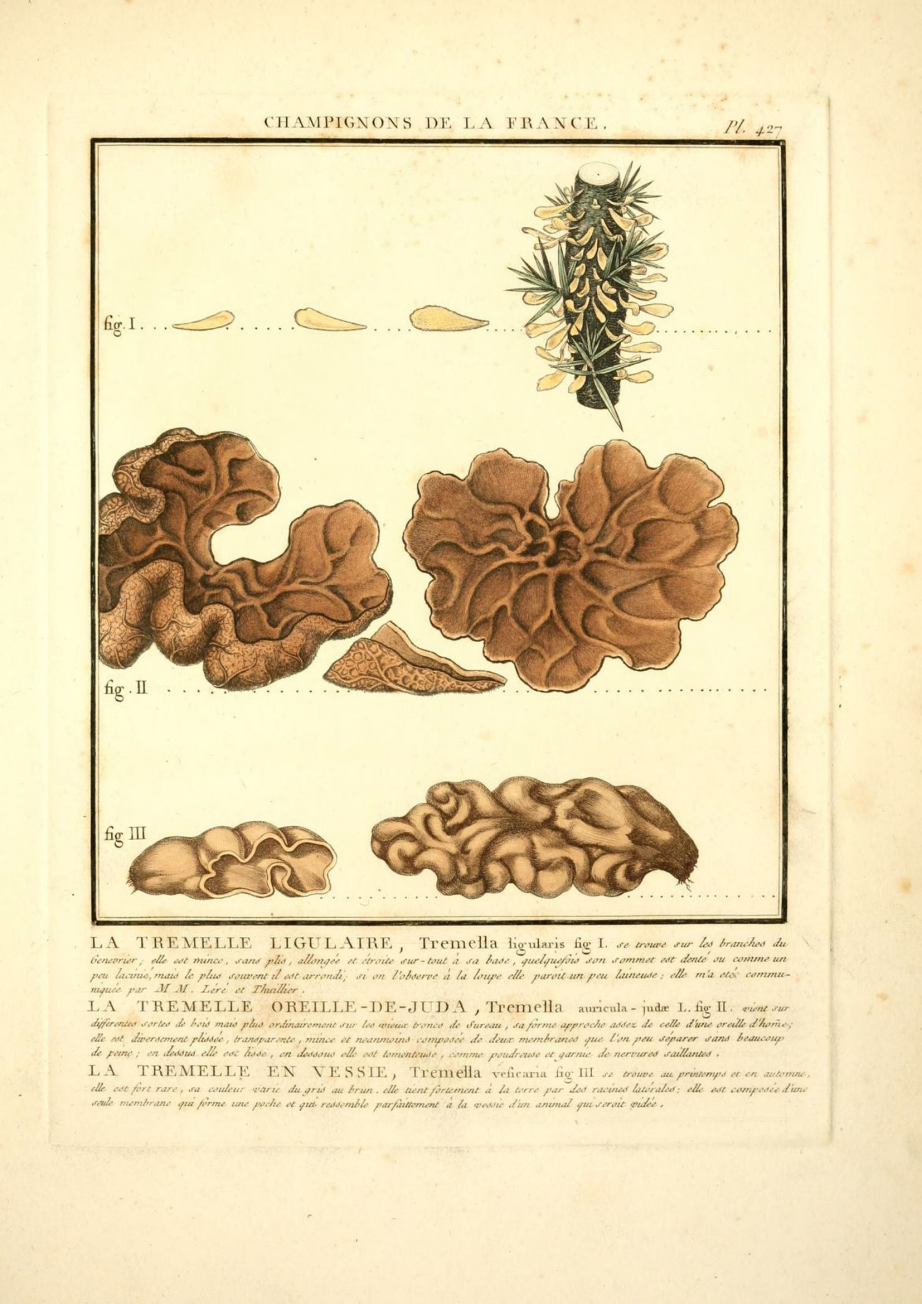
The iconotype painting representing *Tremella auricula-judae* from Jean Baptiste François Pierre Bulliard’s *Herbier de la France* (1789).

A nomenclaturally inconsistent binomial due to name sanctioning isn’t the only issue with the species name as currently designated. When Bulliard established species in *Herbier de la France*, he didn’t designate physical type specimens, rather allowing skillfully rendered image plates to serve as iconotypes ( Figure 3). This means that there is no one individual specimen representing the morphological and genetic qualities used to define *A. auricula-judae*, creating potential for taxonomic confusion. In our modern era, whole genome sequencing allows us to investigate and estimate species boundaries using an abundance of biological information. Without a biological specimen taxonomically tied to a species name, we risk imprecise application of genomic data.

The origins of the name *A. auricula-judae* are complicated, both in publication and in culture. Arguments have been made that “judae” was appended to the specific epithet as a reference to Judas Iscariot, justifying the common name Judas’ ear, from which Jew’s ear would have been bastardized ( Harding 2008). This interpretation relies on several historical references to Judas hanging himself in an elder tree as penance for betraying Jesus ( Knowles 2005) and the observation that *A. auricula-judae* sometimes grows on elder trees ( Kout and Wu 2022). Indeed, Buillard refers to elder trees ( *Sambucus*) in his description: “vient sur différentes sortes de bois mais plus ordinairement sur les vieux troncs de Sureau.”

A dictionary of folk etymology published in 1882 states that the name “Jew’s ear”, describing an ear-like fungus, was a corruption of “Judas’ ear” ( Palmer 1882). However, *The Herball*, printed in 1597, contains the usage of “Iewe’s eare” to describe a wood-dwelling mushroom, with no mention of Judas ( Gerard 1597). *The Regiment of Life,* published even earlier in 1544, also references mushrooms called “iewes eares” ( Goeurot and Phayer 2011). If Jew’s ear is a mere bastardization and the original intention was to associate the fungus with Judas, the corruption must have occurred quite early, persisting until the present. It’s also interesting to note that when Bulliard published the name *Tremella auricula-judae* in 1789, he lists a common name “LA TREMELLE OREILLE-DE-JUDA”—ear of “Juda”, but not Judas specifically.

In French, Judas is spelled exactly the same as it is in English, so did Buillard make an orthographic error with the common name, or was he referring to something else? The French “Juda” can be translated as “Judah.” One meaning of Judah is the Kingdom of Judah, an Israelite Kingdom in the Iron Age centered around Jerusalem and from which Jewish people are primarily descended. Thus “Judah” could be interpreted as interchangeable with “Jew.” However, the translation of the French “Juda” is not entirely clear. Interestingly, the definition for the heading “Juda” in a 1789 French dictionary ( Dictionnaire de l’Académie françoise 1798) is “Ouverture pratiquée à un plancher, et communément fermée d’ une petite trappe amovible, pour voir ce qui se passe au-dessous.”, translated into English as “Opening made in a floor, and commonly closed with a small removable trapdoor, to see what is happening below.” Could Buillard have been simply referring to a peephole? Yet, dictionary entries under various headings from 1694 to 1932 also refer to “les Rois de Juda” or “la tribu de Juda,” clearly demonstrating the well-established use of the term “Juda” in direct reference to Jewish people. It seems likely that, given the prevalence of antisemitic attitudes throughout Europe in the 18th Century, Buillard’s emendment of the Linnaen epithet was intentionally referring to Jewish people and reflects a widely held prejudice of the time. Perhaps Buillard’s embellishment was inspired by his resistance to the emerging tolerance espoused by the revolutionaries that overthrew the French monarchy in the same year *Herbier de la France* was published.

Even if we accept the premise that the added “judae” is a reference to Judas, not the Jewish people, the distinction obviously isn’t clear, judging by the many recorded instances of the name Jew’s ear over several centuries, as well as its use in guidebooks from the 20th century. Author Patrick Harding, who argues that a change to the common name would be the “result of political correctness where it is not necessary”, goes on to say: “The name Jew’s ear is a reminder of the folklore surrounding Judas (himself a Jew …”, revealing that, to Harding, even when the ear is Judas’, it’s still relevant that it’s the ear of a Jew.

Google Scholar lists multiple papers with “Jew’s ear” in their titles published in 2020 and later focused on members of the genus *Auricularia* ( Ekowati *et al*. 2020; Islam *et al*. 2021; Pumnuan *et al*. 2021; Vyshnavi and Pramod 2022; Xu *et al*. 2020). Whether or not one finds this common name offensive, it has become unusual to call a taxon by a name that references an ethnic group. For example, the International Ornithological Community World Bird List has updated several common names of South African birds from previous names that incorporated the term “Hottentot”, a derogatory name addressing the Khoikhoi indigenous group ( Driver and Bond 2021). While this proposal does not recommend any kind of universal rules for avoiding future offensive scientific names or replacing existing ones, it’s an issue worth considering when creating or altering nomenclature. Social and scientific concerns may intersect: organisms with names that are associated with sensitive, controversial, or offensive subject matter might be avoided as research topics, biasing or impeding scientific progress.

Name conservation has been proposed for several fungal taxa to accommodate for discrepancies between the valid binomial and the most commonly used binomial for a species. This issue is most heightened in medicine, where confusion over the identity of organisms could have significant consequences to the ability to treat patients. The name *Cryptococcus gattii* (Vanbreus. and Takashio) Kwon-Chung and Boekhout was conserved against *Cryptococcus hondurianus* Castell. under Article 14 of the *International Code of Nomenclature for algae, fungi, and plants* after *Cryptococcus neoformans* var. *gattii* Vanbreus. and Takashio was raised to species level ( Kwon-Chung *et al*. 2002). Although *C. hondurianus* had priority, the name was not in use within the research community, while *C. gattii* was already widely used and its adoption would minimize difficulties in scientific communication.

Similarly, the phytopathogens *Balansia claviceps* Speg. and *Claviceps paspali* F. Stevens & J.G. Hall and the entomopathogen *Tolypocladium inflatum* W. Gams were all proposed for name conservation by Rossman *et al*. (2017) under Article 14. For each of these taxa, the oldest epithet in the first genus named is valid, but a different epithet has come into common use and a return to the oldest epithet would be disruptive. Name conservation was especially pertinent for *T. inflatum* due to its medicinally important status as the natural source of the immunosuppressant drug cyclosporin. Article 14 of the *Code* deals with name conservation and allows for nomenclatural stability to take precedence over nomenclatural priority were approved by the General Committee.

Although *Auricularia auricula-judae* is undeniably frequently used in scientific and popular mycological literature, *A. auricula* may be used more often, based on a Google Scholar search for “auricularia auricula-judae” returning ~5,050 results, whereas “auricularia auricula -judae” returns ~7,510 results (placing a hyphen, preceded by a space, in front of a term excludes it from Google search results). In our opinion, this unusual, hyphenated epithet, commonly informally or accidentally abbreviated, presents a similar case to name conservations described above.

## Conclusions

We propose that *Auricularia auricula-judae* be returned to the original epithet designated by Linnaeus in *Species Plantarum*, creating the binomial *Auricularia auricula*. *A. auricula* is less controversial, less cumbersome, and already somewhat in use as an invalid synonym published in *Mycologia* by Bernard Lowy in 1952 ( Lowy 1952). We also suggest designating a physical epitype in addition to the existing iconotype illustration created by Bulliard in *Herbier de la France* ( Figure 3). Our recommendation is that an epitype specimen should be selected from the dataset of Wu *et al*. (2021), which delineated species boundaries using a multigene phylogeny.

## Data Availability

No data are associated with this article.

## References

[ref1] Arora D : *All that the rain promises and more: a hip pocket guide to western mushrooms.* Ten Speed Press;1991.

[ref2] Dictionnaire de l’Académie françoise [Texte imprimé], revu, corrigé et augmenté par l’Académie elle-même: À Paris, chez J. J. Smits et Ce. imp.-lib. 5th ed. 1798.

[ref3] Driver RJ Bond AL : Towards redressing inaccurate, offensive and inappropriate common bird names. 2021.

[ref4] Ekowati N Maharning AR Ratnaningtyas NI : Effects of Ethyl Acetate Extract of Jew’s Ear Mushrooms (Auricularia auricula) on Cytotoxic and Apoptosis of Cervical Cancer Cells (HeLa). *IOP Conference Series: Earth and Environmental Science.* IOP Publishing;2020, November; (Vol.593(1): p.012011).

[ref5] Fries E : *Systema mycologicum: Sistens fungorum ordines, genera et species, huc usque cognitas, quas ad normam methodi naturalis determinavit.* Lundae, Ex Officina Berlingiana;1821; (Vol.1). 1821-[1832]. Reference Source

[ref25] Gerard J Dodoens R Priest R : *The Herball, or, Generall historie of plantes. Imprinted at London.* Iohn Norton;1597. Reference Source

[ref6] Goeurot J Phayer T : *The regiment of life, whereunto is added a treatise of the pestilence, with the boke of children, newly corrected and enlarged by T. Phayre.* Houssemaine, Nicolas de, d. 1523. Early English Books Online Text Creation Partnership. 2011. accessed 30 March 2023. Reference Source

[ref7] Harding P : *Collins Mushroom Miscellany.* Collins;2008. Reference Source

[ref8] Herbier de la France: ou, Collection complette des plantes indigenes de ce royaume; avec leurs proprie ´te ´s, et leurs usages en medecine, Paris, Chez l’auteur, Didot, Debure, Belin. 1780-93. Reference Source

[ref9] Islam T Ganesan K Xu B : Insights into health-promoting effects of Jew’s ear (Auricularia auricula-judae). *Trends Food Sci. Technol.* 2021;114:552–569. 10.1016/j.tifs.2021.06.017

[ref10] Knowles E : *The Oxford Dictionary of Phrase and Fable.* Oxford University Press;2005. Retrieved 1 May. 2023. 10.1093/acref/9780198609810.001.0001/acref-9780198609810

[ref11] Kout J Wu F : Revealing the Cryptic Diversity of Wood-Inhabiting Auricularia (Auriculariales, Basidiomycota) in Europe. *Forests.* 2022;13(4). Article 4. 10.3390/f13040532

[ref12] Kwon-Chung KJ Boekhout T Fell JW : (1557) Proposal to Conserve the Name Cryptococcus gattii against C. hondurianus and C. bacillisporus (Basidiomycota, Hymenomycetes, Tremellomycetidae). *Taxon.* 2002;51(4):804–806. 10.2307/1555045

[ref13] Linné C von : *Species plantarum: Exhibentes plantas rite cognitas ad genera relatas, cum diferentiis specificis, nominibus trivialibus, synonymis selectis, locis natalibus, secundum systema sexuale digestas:* Vol. t.1 (1753). Berlin, Junk, 1908. 1753. Reference Source

[ref14] Lowy B : The Genus Auricularia. *Mycologia.* 1952;44(5):656–692. Reference Source

[ref15] May TW Redhead SA Bensch K : Chapter F of the *International Code of Nomenclature for algae, fungi, and plants* as approved by the 11th International Mycological Congress, San Juan, Puerto Rico, July 2018. *IMA Fungus.* 2019;10:21. 10.1186/s43008-019-0019-1 32647625 PMC7325661

[ref16] Palmer AS : *Folk-etymology: A Dictionary of Verbal Corruptions Or Words Perverted in Form Or Meaning, by False Derivation Or Mistaken Analogy.* G. Bell and Sons;1882. Reference Source

[ref17] Pumnuan J Insung A Phutphat S : Carbaryl Insecticide Decomposition after Application on Oyster and Jew’s Ear Mushrooms Cultivations. *Science & Technology Asia.* 2021;169–175.

[ref18] Quélet L : *Enchiridion fungorum in Europa media et praesertim in Gallia vigentium.* O. Doin;1886. Reference Source

[ref19] Rossman AY Allen WC Castlebury LA : (2517–2519) Proposals to conserve the names Balansia claviceps against Ephelis mexicana, Claviceps paspali against Ustilagopsis deliquescens, and Tolypocladium inflatum against Cordyceps subsessilis (Ascomycota: Sordariomycetes: Hypocreales). *Taxon.* 2017;66(3):749–750. 10.12705/663.18

[ref20] Smith AH : *A Field Guide to Western Mushrooms.* University of Michigan Press;1975. Reference Source

[ref21] Vyshnavi AS Pramod R : Effect of supplements and growth regulators on the productivity of Jew’s ear mushroom (Auricularia auricula-judae). *The Pharma Innovation Journal.* 2022;11(12):4261–4265.

[ref22] Wu F Tohtirjap A Fan LF : Global Diversity and Updated Phylogeny of *Auricularia* (Auriculariales, Basidiomycota). *J. Fungi (Basel).* 2021 Nov 3;7(11):933. 10.3390/jof7110933 34829220 PMC8625027

[ref23] Wu F Yuan Y He S-H : Global diversity and taxonomy of the Auricularia auricula-judae complex (Auriculariales, Basidiomycota). *Mycol. Prog.* 2015;14(10). 10.1007/s11557-015-1113-4

[ref24] Xu Z Meenu M Xu B : Effects of UV-C treatment and ultrafine-grinding on the biotransformation of ergosterol to vitamin D2, physiochemical properties, and antioxidant properties of shiitake and Jew’s ear. *Food Chem.* 2020;309:125738. 10.1016/j.foodchem.2019.125738 31706679

