# Supplementary material for: Whose Ear?: Proposal to conserve the name Auricularia auricula (L.) Underw. for Auricularia auricula-judae (Bull.) Quél
Source: F1000Res. 2023 Aug 7;12:948. [Version 1] doi: 10.12688/f1000research.134821.1 (PMC11009569; doi:10.12688/f1000research.134821.1)
Supplement: Peer review history

# Reviewer report: Reviewer response for version 1

**DOI:** 10.5256/f1000research.147906.r261262

**Published:** 2024-04-11

**Authors:** Nattapol Kraisitudomsook

**Affiliations:** 1 Biology, Muban Chombueng Rajabhat University, Chom Bueng District, Ratchaburi, Thailand

Major comments:

 The authors did an excellent job weaving a story about *Auricularia auricula-judae *and its problematic epithet. I applaud the authors initiative to take actions combating racism and antisemitism. And I am totally on board with having an actual fungarium specimen as epitype. I only have a few concerns and questions below.

 I think another possible reason for Bulliard to emend the name is to avoid taxonomic confusion. Has the authors looked to make sure that there were not two separate *Tremella auricula* species descriptions around 1789?

 And if Buillard really added -judae to refer to the Jews, then I wonder. What about this fungus made him think of the Jewish people? As a non-Western descent, I am only aware of the red curly hair and the hooked nose stereotypes. Did he think that Jews had venous ears or something similar to *A. auricula*? I think it would be a nice addition to this article if the authors researched and discussed this part. 

 Lastly, for me, the main reason to conserve a scientific name should be for clarity and usage. In this case, auricula-judae is also commonly used (5,000 results is a lot) to represent the wood ear mushroom. On one hand, it is preferred to have a consistent scientific name. On the other hand, the proposed conservation might be disruptive to researchers who are currently using 'auricularia-judae' to talk about this fungus. So usually I would be on the fence about this proposal. However, with added antisemitic connotation, I favor the conservation of the name *Auricularia auricula*. 

 Minor comments:

 1. For "antisemitic attitudes throughout Europe in the 18th Century...and reflects a widely held prejudice of the time" - this line might need a citation as it should be a historical fact. 

 2. For "auricularia auricula -judae returns ~7,510 results" - I understand what you did, but it might be confusing to an average reader because the two names look very similar. Why not just say "auricularia auricula" returns about 7,500 results. The authors don't have to go into a technical detail on how to exclude -judae in Google Scholar searches.

 3. For "lists only Auricularia auricula ( Smith 1975)." the name should be italicized.

Is the topic of the opinion article discussed accurately in the context of the current literature?

Yes

Are arguments sufficiently supported by evidence from the published literature?

Yes

Are all factual statements correct and adequately supported by citations?

Yes

Are the conclusions drawn balanced and justified on the basis of the presented arguments?

Yes

Reviewer Expertise:

wood-decay fungi taxonomy, evolution, and systematics

I confirm that I have read this submission and believe that I have an appropriate level of expertise to confirm that it is of an acceptable scientific standard.

# Reviewer report: Reviewer response for version 1

**DOI:** 10.5256/f1000research.147906.r245333

**Published:** 2024-02-23

**Authors:** Rong-Ju Xu

**Affiliations:** 1 School of Science, Mae Fah Luang University, Mueang Chiang Rai, Chiang Rai, Thailand

This manuscript, titled "Whose Ear?: Proposal to conserve the name *Auricularia auricula* (L.) Underw. for *Auricularia auricula-judae* (Bull.) Quél.", explores the nomenclatural and cultural history of the fungus *Auricularia auricula-judae*, advocating for a return to its original specific epithet " *auricula*". The authors provide a thorough examination of the historical, cultural, and scientific aspects surrounding the naming of this species. They argue that the name change to " *auricula-judae*" does not conform to standard nomenclatural practices and that the original name " *auricula*" should be restored to reduce confusion and align with modern taxonomy principles. Additionally, they propose designating a physical epitype to provide clarity and stability to the species taxonomy.

   Overall, the paper presents a compelling argument for revisiting the nomenclature of *Auricularia auricula-judae*, blending scientific rigour with cultural and historical awareness.

Is the topic of the opinion article discussed accurately in the context of the current literature?

Yes

Are arguments sufficiently supported by evidence from the published literature?

Yes

Are all factual statements correct and adequately supported by citations?

Yes

Are the conclusions drawn balanced and justified on the basis of the presented arguments?

Yes

Reviewer Expertise:

Phylogenetics, Taxonomy

I confirm that I have read this submission and believe that I have an appropriate level of expertise to confirm that it is of an acceptable scientific standard.
